# The global molecular epidemiology of microsporidia infection in sheep and goats with focus on *Enterocytozoon bieneusi*: a systematic review and meta-analysis

**DOI:** 10.1186/s41182-021-00355-7

**Published:** 2021-08-24

**Authors:** Ali Taghipour, Saeed Bahadory, Ehsan Javanmard

**Affiliations:** 1 Zoonoses Research Center, Jahrom University of Medical Sciences, Jahrom, Iran; 2 Department of Parasitology, Faculty of Medical Sciences, Tarbiat Modares University, Tehran, Iran; 3 Department of Medical Parasitology and Mycology, School of Public Health, Tehran University of Medical Sciences, Tehran, Iran

**Keywords:** Microsporidia, *Enterocytozoon bieneusi*, Sheep, Goat, Meta-analysis

## Abstract

**Background:**

Microsporidia is a zoonotic pathogen with health consequences in immunocompromised patients. Small ruminants are a potential reservoir of microsporidia for humans in their vicinity. Hence, we aimed to evaluate the molecular prevalence of microsporidian infections with emphasis on *Enterocytozoon bieneusi* genotypes among sheep and goats at a global scale through systematic review and meta-analysis approach.

**Methods:**

The standard protocol of preferred reporting items for systematic reviews and meta-analyses (PRISMA) guidelines were followed. Eligible prevalence studies on small ruminant microsporidiosis, published from 1 January 2000 until 15 April 2021 were gathered using systematic literature search in PubMed, Scopus, Web of Science and Google Scholar databases. Inclusion and exclusion criteria were applied. The point estimates and 95% confidence intervals were calculated using a random-effects model. The variance between studies (heterogeneity) was quantified by *I*^2^ index.

**Results:**

In total, 25 articles (including 34 datasets) were included for final meta-analysis. The pooled molecular prevalence of microsporidia in sheep and goats was estimated to be 17.4% (95% CI: 11.8–25%) and 16% (95% CI: 11.2–22.4%), respectively. Likewise, the overall prevalence of *E. bieneusi* was estimated to be 17.4% (95% CI: 11.8–25%) for sheep and 16.3% (95% CI: 11.3–22.8%) for goats. According to internal transcribed spacer (ITS) gene analysis, *E. bieneusi* with genotypes BEB6 (15 studies) and COS-1 (nine studies) in sheep, and CHG3 (six studies) and BEB6 (five studies) in goats were the highest reported genotypes.

**Conclusion:**

The present results highlight the role of sheep and goats as reservoir hosts for human-infecting microsporidia. Therefore, this global estimate could be beneficial on preventive and control measures.

**Supplementary Information:**

The online version contains supplementary material available at 10.1186/s41182-021-00355-7.

## Introduction

Microsporidia are a diverse group of zoonotic pathogens parasitizing invertebrates (insects) and vertebrates (fish, birds and mammals) [1]. *Enterocytozoon bieneusi* and *Encephalitozoon* spp. (i.e., *Enc. intestinalis*, *Enc. hellem*, and *Enc. cuniculi*) are two well-known genera among microsporidian species [2], with *E. bieneusi* being responsible for over 90% of animal and human cases [3]. A distinctive stage in the microsporidian life cycle is the formation of infective spores, which potentially contaminate the environment including water supplies and foodstuff [4–6]. Clinical infection is frequently eminent in immunocompromised patients, manifesting as malabsorption with subsequent chronic diarrhea as well as wasting diathesis [7, 8]. Additionally, microsporidian infections in immunocompetent subjects are asymptomatic but important, since these individuals are carriers of infective spores as a significant epidemiological concern [7]. Previously, the global prevalence of microsporidia infections was estimated among HIV-positive patients, rendering a 11.8% (95% CI: 10.1–13.4%) pooled prevalence [9]. A considerably high total prevalence of microsporidia infection was, also, calculated among cat populations worldwide [29.7% (95% CI: 19.7–42.2%)] [10], rather than in dogs [23.1% (95% CI: 13.5–36.8%)] [11]. As mentioned previously, *E. bieneusi* is the most prevalent genus among other microsporidian species, which demands molecular approaches to be exactly identified and genotyped [12]. Molecular techniques based on the variations in the nucleotide sequence of the internal transcribed spacer (ITS) region of the rRNA gene are mostly preferred for the identification of *E. bieneusi* genotypes [12]. Until now, over 200 distinct genotypes of *E. bieneusi* have been identified in humans, animals or both [13]. Small ruminants (sheep and goat) contribute a major role in the production of various dairy products worldwide [14, 15]. Diarrhea is a common intestinal sequela of microsporidian infections in small ruminants, causing considerable mortality and production loss [5, 16]. As well, there are some reports of raw milk contamination by microsporidian agents in sheep and goats [5, 17]. However, little is known on the molecular prevalence and genotype distribution of microsporidia, particularly *E. bieneusi* genotypes, in small ruminants. Thereby, the present systematic review and meta-analysis was done to evaluate the molecular prevalence of microsporidian infections with emphasis on *Enterocytozoon bieneusi* genotypes among sheep and goats at a global scale.

## Methods

### Information sources and systematic search

The present systematic review and meta-analysis was performed based on the preferred reporting items for systematic reviews and meta-analyses (PRISMA) statement [18]. Four international databases (PubMed, Scopus, Web of Science and Google Scholar) were excavated to gather relevant records on the molecular prevalence of microsporidia infection in sheep and goats published between 1 January 2000 and 15 April 2021. The search process was accomplished using MeSH terms alone or in combination: (Microsporidium” OR “Microsporidia” OR “Microspora” OR “*Enterocytozoon bieneusi*” OR “*Encephalitozoon* spp.”) AND (“Prevalence” OR “Epidemiology”) AND ("Small Ruminant" OR "Sheep” OR “Goat”). In addition, the bibliographic list of initially found papers was manually searched to find other relevant citations.

### Inclusion criteria, study selection and data extraction

The inclusion criteria for the present systematic review were as follows: (1) abstracts and/or full-texts published in English language; (2) cross-sectional original papers or short reports estimating the molecular prevalence of microsporidia infection in sheep and goats; (3) utilization of different molecular methods; (4) papers providing total sample size and positive samples; and (5) published online from 1 January 2000 until 15 April 2021. Two independent reviewers evaluated the articles based on determined inclusion criteria and possible contradictions in cases of study selection or extraction procedure were obviated by discussion and consensus. Also, those articles on microsporidia infection in humans or other animals, studies that used non-molecular diagnostics, experimental investigations in small ruminants, as well as review papers, cohort, case-reports, case series, and editorials were all excluded. In the following, a set of required information, including first author’s last name; year of publication; continent; country; small ruminant species (sheep or goats); number of examined animals; number of animals with a positive test result, age, gender, molecular methods, identified parasite species and gene targets were precisely extracted.

### Study quality assessment

The Joanna Briggs Institute (JBI) checklist is a qualitative index for inclusion of articles [19], providing ten questions with four options including, Yes, No, Unclear, and Not applicable. Briefly, a study can be awarded a maximum of one star for each numbered item. Those papers with a total score of 4–6 and 7–10 points were assigned as moderate and high quality, respectively.

### Meta-analysis

The comprehensive meta-analysis Bio stat v2.2 software was employed for meta-analysis procedure [10, 11, 20]. Calculation of the pooled prevalence of microsporidia infection among small ruminants and 95% confidence intervals (CIs) was done using random-effects model (REM), which enhances the distribution of true effect sizes among studies [21, 22]. Subgroup analysis was, also, performed in order to reveal the weighted prevalence based on continent, country, and type of ruminants (sheep and goats). Moreover, the probable association of microsporidia prevalence with age and gender was determined using REM-based odd ratio (OR) estimation. The heterogeneity between studies was computed via *I*^*2*^ index and the Cochrane’s *Q* statistics [10, 11, 23]. Funnel plot was used to show the probability of publication bias [24]. Forest plot diagram was utilized to represent the pooled prevalence (with 95% CI) of microsporidia infection in sheep and goats.

## Results

**Fig. 1:**
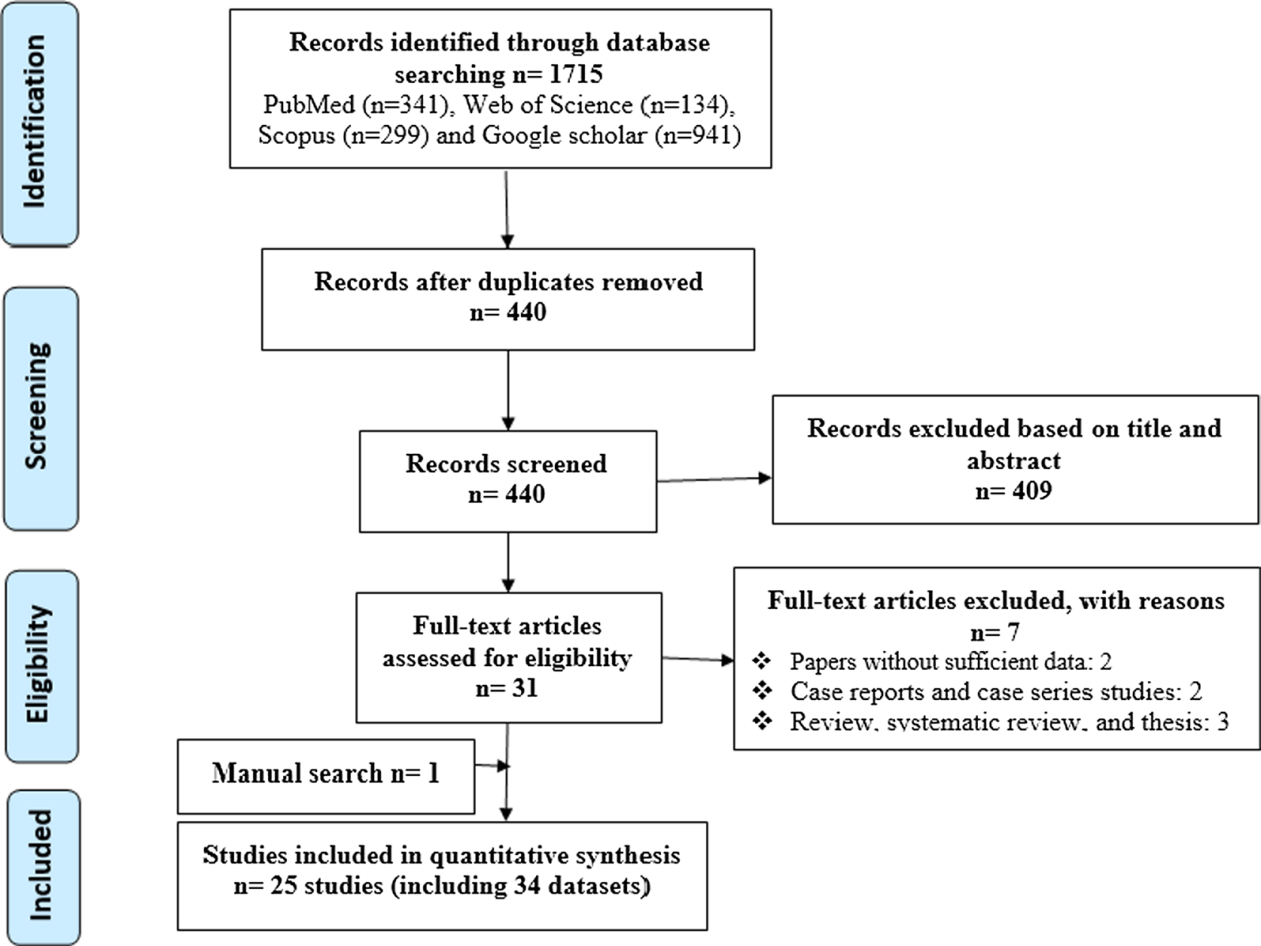
PRISMA flow diagram describing included/excluded studies

Following comprehensive systematic search (Fig. 1), 1715 records were initially retrieved, among which many duplicate/non-eligible articles were removed and only 25 papers were finally eligible to undergo meta-analysis [16, 25–48]. Of note, 9 out of 25 studies possessed more than one dataset (Table 1), so that 34 datasets (20 datasets for sheep and 14 for goat) were reviewed and required data were extracted. Table 1 shows the results of the quality assessment based on the JBI checklist, rendering acceptable quality for all articles.

**Table 1:** All the studies investigating the global prevalence of microsporidia species in sheep and goats according to molecular methods

First author/continent/countries	Publication year	Sample type	Diagnostic method	Gene	Animal	Sample size (*n*)	Infected by microsporidia (*n*)	*E. bieneusi* (*n*)	*Enterocytozoon bieneusi* (genotypes (n; number))	QA
America										
Brazil										
da Silva Fiuza et al. [33]	2016	Fecal	Nested-PCR	ITS	Sheep	125	24	24	BEB6 (11), BEB7 (8), I (2),BEB18 (1), BEB19 (1), and LW1 (1)	10
Asia										
China										
Ye et al. [27]	2015	Fecal	Nested-PCR	ITS	Sheep	375	260	260	BEB6(237), and CM7(23)	10
Zhang et al. [48]	2020	Fecal	Nested-PCR	ITS	Goat	300	89	89	Unknown	8
Zhang et al. [48]	2020	Fecal	Nested-PCR	ITS	Sheep	360	148	148	Unknown	8
Qi et al. [28]	2019	Fecal	Nested-PCR	ITS	Sheep	318	20	20	BEB6 (12), CHG1 (1), CHG3 (1), CHS3 (1), CHS8 (1), COS-I (2), XJS1 (1), and XJS2(1)	9
Chang et al. [31]	2019	Fecal	Nested-PCR	ITS and SSU rRNA	Sheep	620	93	93	BEB6 (6), CHC8 (68), CHG1 (1), CHG19 (7), H (2), I (1), CTS1 (1), CTS2 (2), PigEBITS5 (3), and CTS3 (1)	10
Chang et al. [31]	2019	Fecal	Nested-PCR	ITS and SSU rRNA	Goat	260	25	25	EbpA (15) and EbpC (16)	10
Chen et al. [32]	2018	Fecal	Nested-PCR	ITS	Sheep	325	40	40	BEB6 (24), COS I (2), COS II (1), CHG1 (1), and YSM1 (1)	8
Chen et al. [32]	2018	Fecal	Nested-PCR	ITS	Goat	336	30	30	BEB6 (10), COS I(4), SX1 (3), CM21 (2), CHG3 (2), PigEb4 (1), CHS5 (1), EbpC (1), and YNS1 (1)	8
Li et al. [35]	2019	Fecal	Nested-PCR	ITS	Sheep	832	28	28	COS-I (6), OEB1 (3), BEB6 (9), CHC8 (3), AHS1 (4), AHS2 (2), and JSS1 (1)	9
Li et al. [35]	2019	Fecal	Nested-PCR	ITS	Goat	781	32	32	CHG1 (12), CHG3 (15), AHG1 (1), AHG2 (2), BEB6 (1), and COS-II (1)	9
Zhou et al. [37]	2019	Fecal	Nested-PCR	ITS	Goat	341	82	82	CHG5 (47), CHG3 (23), CHG2 (4), CM21 (3), D (2), AHG1 (1), HNG-I (1), and HNG-II (1)	10
Peng et al. [38]	2016	Fecal	Nested-PCR	ITS and SSU rRNA	Goat	629	179	179	BEB6 (51), CHS7 (14), CHG1 (42), CHG2 (2), SX1 (56), and COSI (14)	9
Peng et al. [39]	2019	Fecal	Nested-PCR	ITS	Sheep	1014	124	124	BEB6 (111), COS-I (3), CHG13 (3), NX1 (1), NX2 (1), NX3 (1), NX4 (1), NX5 (1), NX6 (1), NX7 (1)	10
Li et al. [40]	2014	Fecal	Nested-PCR	ITS	Sheep	45	2	2	BEB6 (2)	9
Yang et al. [42]	2018	Fecal	Nested-PCR	ITS	Sheep	953	194	194	BEB6 (129), CHS8 (32), CHG1 (14), CHG3 (5), CHS7 (3), COS-I (3), CHHLJS1 (3), CHHLJS2 (2), CHNXS1 (1), CHXJS1 (1), and NESH5 (1)	10
Shi et al. [16]	2016	Fecal	Nested-PCR	ITS	Goat	611	176	176	BEB6(41), D(3), E(8), F(4), KIN-1(2), J(1), CHG1(19), CHG2(6), CHG3(17), CD6(9), CHG5(7), CHG6(1),CHG7(1), CHG8(1), CHG9(1), CHG10(1), CHG11(1), CHG12(1), CHG13(1), CHG14(1), COS-I(2), CHG16(2), CHG17(1), CHG18(1), CHG19(1), CHG20(1), CHG21(1), CHG22(1), CHG23(1), CHG24(1), and CHG25(1)	9
Shi et al. [16]	2016	Fecal	Nested-PCR	ITS	Sheep	414	177	177	BEB6(60), COS-I(14), CHG3(5), CM4(1), CHS3(2), CHS4 (1), CHS5 (1), CHS6 (1), CHS7 (1), CHS8 (1), CHS9(1)CHS10 (1), CHS11(1), and CHS12 (1)	9
Wu et al. [43]	2018	Fecal	Nested-PCR	ITS and SSU rRNA	Sheep	177	61	61	CM7 (30), BEB6 (14), CHS3 (2), CM7 (4), BEB6 (8), and CGS1(3)	10
Zhang et al. [44]	2018	Fecal	Nested-PCR	ITS	Sheep	312	73	73	BEB6 (31), COS-I (25), NESH5 (11), CHS17 (2), CHS13 (1), CHS14 (1), CHS15 (1), and CHS16 (1)	10
Zhang et al. [45]	2019	Fecal	Nested-PCR	ITS and SSU rRNA	Sheep	78	7	7	CHS8 (3) and COS I (4)	9
Zhang et al. [45]	2019	Fecal	Nested-PCR	ITS and SSU rRNA	Goat	59	11	11	CHG2 (9) and CHG3 (2)	9
Zhao et al. [46]	2015	Fecal	Nested-PCR	ITS	Goat	55	12	12	Peru6 (3), BeB6 (3), D (2), EbpC (2), EbpA (1), and COG-I (1)	9
Zhao et al. [46]	2015	Fecal	Nested-PCR	ITS	Sheep	138	31	31	BeB6 (12), Peru6 (5), D (4), O (3), and COS-I to COS-VII (one each)	9
Jiang et al. [47]	2015	Fecal	Nested-PCR	ITS	Sheep	489	68	68	BEB6 (28), CM7 (3), CS-4 (4), NESH1 to NESH3 (1 each), NESH5 (1), OEB1(3), BEB6/CM7a (5), BEB6/NESH4a (3), BEB6/NESH6a (1), BEB6/OEB1a (5), and CS-4/EbpCa (1)	9
Africa										
Egypt										
*Abu-Akkada et al. [30]	2015	Urine	PCR	SSUrRNA	Goat	40	0			8
**Al-herrawy and Gad [34]	2016	Fecal	PCR	SSU-rRNA	Goat	83	15	11		8
Al-herrawy and Gad [34]	2016	Fecal	PCR	SSU-rRNA	Sheep	89	6	6		8
Asia										
Iran										
Askari et al. [36]	2015	Fecal	Nested-PCR	SSU-rRNA	Sheep	30	3	3		9
Thailand										
Udonsom et al. [41]	2019	Fecal	Nested-PCR	ITS and SSU rRNA	Goat	73	14	14	GoatAYE1(4), H (PEbC) (1), SX1(1),CHC8(4),and CHG3(4)	9
Europe										
Slovakia										
Valencakova and Danisova [25]	2019	Fecal	Real-time SYBR green- PCR	ITS, SSUrRNA	Sheep	33	0	0		8
Valencakova and Danisova [25]	2019	Fecal	Real-time SYBR green-PCR	ITS, SSUrRNA	Goat	20	0	0		8
Spain										
Lores et al. [29]	2002	Fecal	PCR	SSUrRNA	Goat	7	1	1		9
Sweden										
Stensvold et al. [26]	2014	Fecal	Nested-PCR	ITS	Sheep	109	49	49	BEB6 (32), OEB1 (6), OEB2 (2), BEB6 + OEB1 (4), BEB6 + OEB2 (4), and ND (1)	9

*The aim of this study was to detect *Enc. cuniculi*, but no cases were found

**In this study, four cases of *Enc. intestinalis* infection were found

*QA* quality assessment

**Fig. 2:**
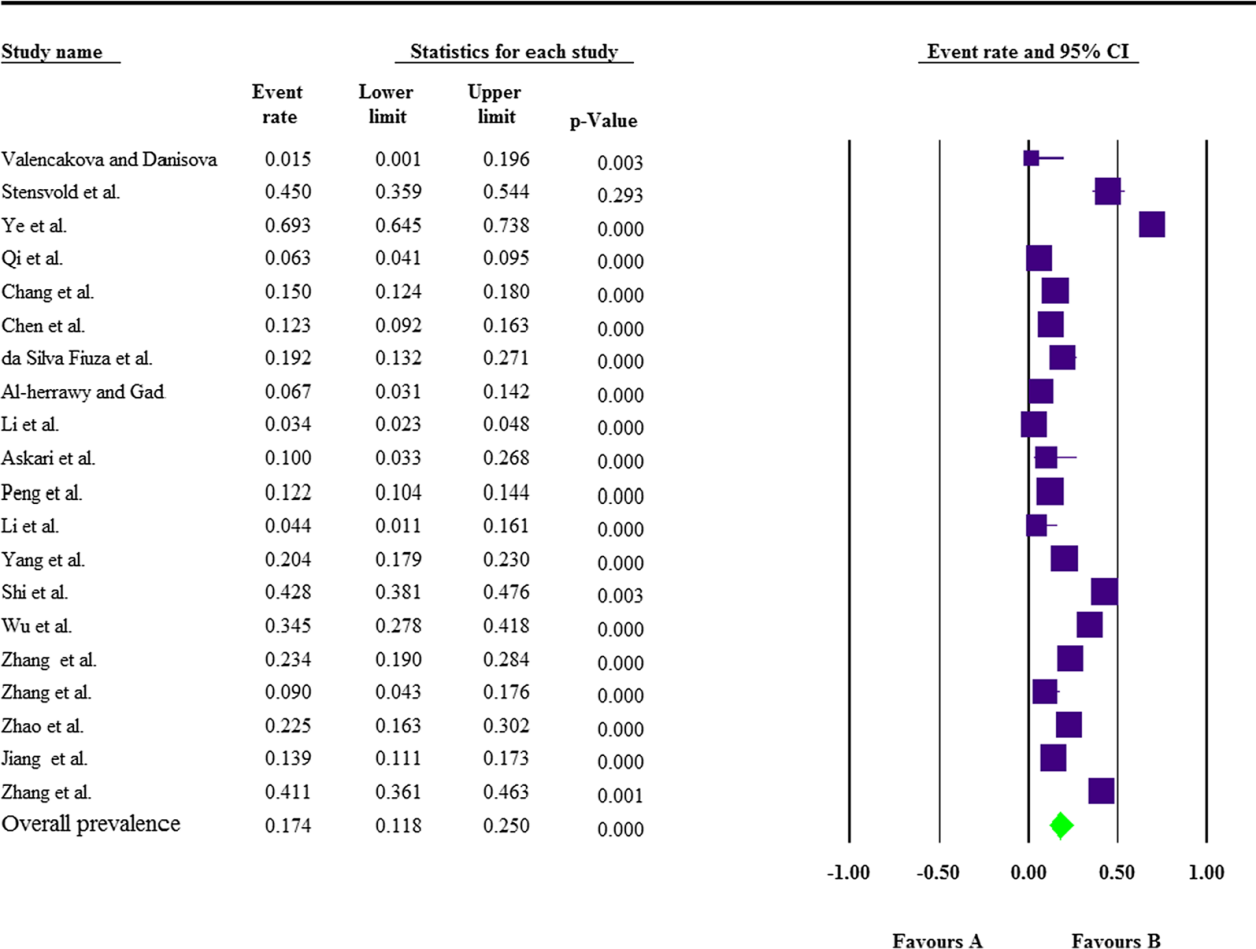
The pooled molecular prevalence of *E. bieneusi* infection in sheep

**Fig. 3:**
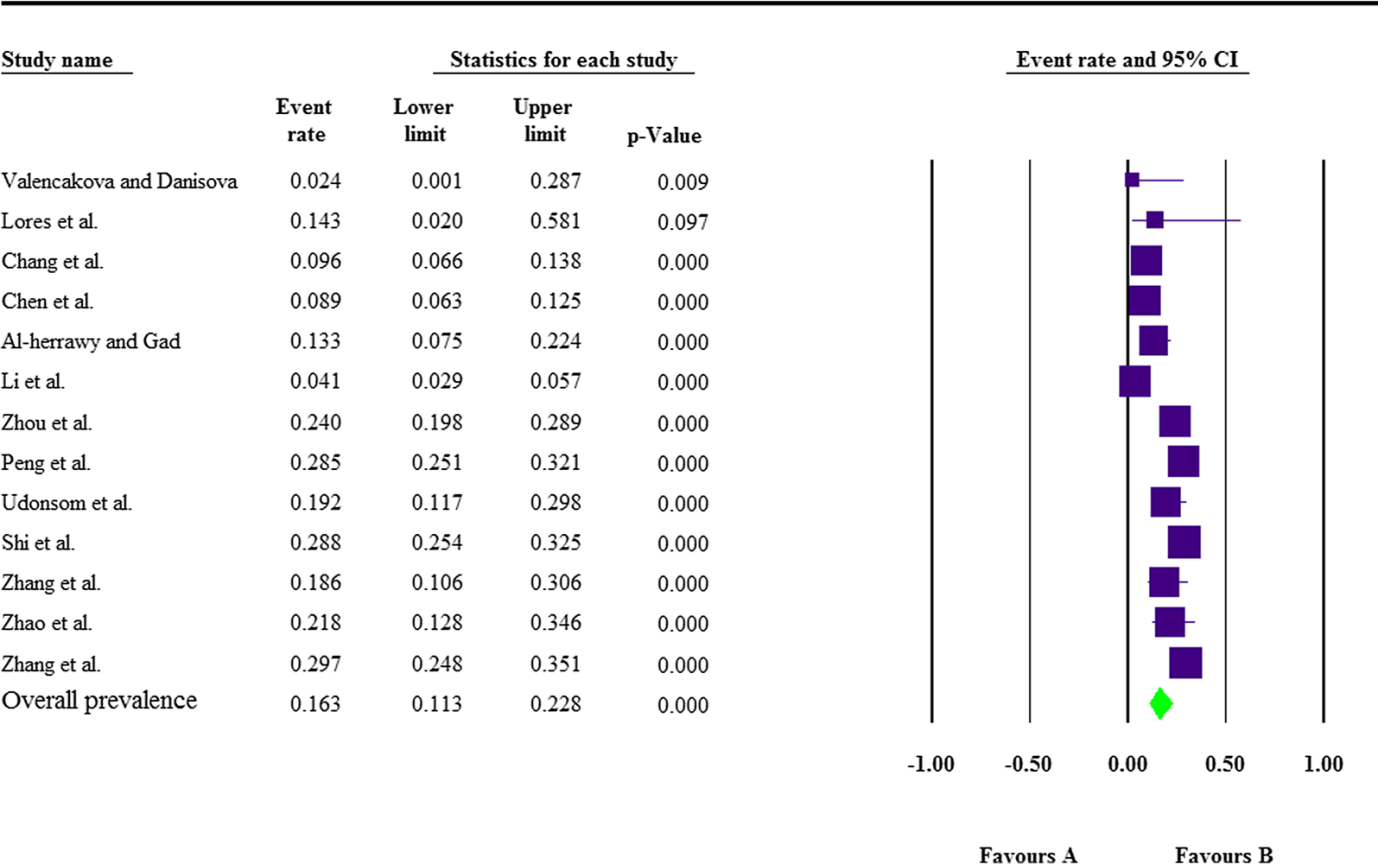
The pooled molecular prevalence of *E. bieneusi* infection in goats

All datasets represented molecular characterization of microsporidia infections in small ruminants from 8 countries located at 4 continents, including Asia (26 datasets, 9925 animals), Europe (four datasets, 169 animals), Africa (three datasets, 212 animals) and America (one dataset, 125 animals) (Tables 1 and 2). China possessed the most published literature with 17 studies and 24 datasets. Most studies focused on *E. bieneusi* and only one study reported *Enc. intestinalis* in goats [34] (Table 1). In addition, one study out of the total study focused only on the detection of *Enc. cuniculi* in goats [30]. A relatively moderate weighted prevalence of microsporidia infection was obtained for both sheep 17.4% (95% CI: 11.8–25%) and goats 16% (95% CI: 11.2–22.4%) (Additional file 1: Figs. S1 and 2). Similar pooled prevalence rates were estimated for *E. bieneusi* in both sheep 17.4% (95% CI: 11.8–25%) (Fig. 2) and goats 16.3% (95% CI: 11.3–22.8%) (Fig. 3). The molecular determination of *E. bieneusi* genotypes was frequently accomplished using ITS gene, and genotypes BEB6 (15 studies) and COS-1 (nine studies) in sheep, and CHG3 (six studies) and BEB6 (five studies) in goats were the most prevalent among all other genotypes (Table 1). America and Asia continents showed the highest total prevalence rates with 19.2% (95% CI: 13.2–27.1%) and 17.6% (95% CI: 13.1–23.3%), respectively, followed by Europe 10.2% (95% CI: 1.4–48.3%), and Africa 8.7% (95% CI: 2.9–23.6%) (Table 2). It is noteworthy that Table 2 demonstrates data on country-based prevalence of microsporidia infection.

**Table 2:** Subgroup analysis of continents, countries and animal type (sheep and goats), based on molecular methods

Variables	Datasets (*n*)	Total samples (*n*)	Infected (*n*)	Pooled prevalence% (95% CI)	Heterogeneity			
Continent/countries					*I*^2^ (%)	*Q*-value	*P*-value	*t*^2^
Africa	3	212	21	8.7% (2.9–23.6%)	74.592	7.871	0.020	0.695
Egypt	3	212	21	8.7% (2.9–23.6%)	74.592	7.871	0.000	0.695
America	1	125	24	19.2% (13.2–27.1%)	0.000	0.000	1.000	0.000
Brazil	1	125	24	19.2% (13.2–27.1%)	0.000	0.000	1.000	0.000
Asia	26	9925	1979	17.6% (13.1–23.3%)	97.517	1007.573	0.000	0.766
China	24	9822	1962	17.9% (13.1–23.8%)	97.708	1003.573	0.000	0.774
Thailand	1	73	14	19.2% (11.7–29.8%)	0.000	0.000	1.000	0.000
Iran	1	30	3	10% (3.3–26.8%)	0.000	0.000	1.000	0.000
Europe	4	169	50	10.2% (1.4–48.3%)	80.248	15.188	0.002	3.466
Slovakia	2	53	0	1.9% (0.3–12.1%)	0.000	0.059	0.808	0.000
Spain	1	7	1	14.3% (2–58.1%)	0.000	0.000	1.000	0.000
Sweden	1	109	49	45% (35.9–54.4%)	0.000	0.000	1.000	0.000
Animal type								
Sheep	20	6836	1401	17.4% (11.8–25%)	97.747	843.168	0.000	0.970
Goat	14	3595	666	16% (11.2–22.4%)	93.902	213.175	0.000	0.479

A positive association was observed between microsporidia infection with age (≤ 3 months) (OR = 2.044; 95% CI, 1.35–3.093%) and male gender (OR = 3.169; 95% CI, 2.215–4.535%) (Table 3). The included studies had a significant publication bias based represented in the funnel plot (Additional file 1: Fig. S3 for sheep and Additional file 1: Fig. S4 for goats).

**Table 3:** Gender and age associated with microsporidia infection among sheep and goats worldwide

Risk factors	Datasets (*n*)	Variables	Total samples (*n*)	Infected samples (*n*)	Pooled prevalence% (95 CI)	OR (95% CI)	OR heterogeneity (*I*^2^%)
Gender	2	Male	276	68	24.7% (19.9–30.1%)	3.169 (2.215–4.535%)	0.000
		Female	863	80	11.2% (6–19.8%)		
Age	8	≤ 3 months	436	212	49.6% (28.1–71.2%)	2.044 (1.35–3.093%)	67.821
		> 3 months	1018	318	25.6% (13.3–43.6%)		

## Discussion

The health of animals and human are tightly interconnected within the environmental context, what is called as the One Health approach [49]. Domestic animals such as sheep and goats are in close contact with humans in rural areas and may contribute to some zoonotic pathogens including microsporidia infections [46]. Hence, a global evaluation of the pooled prevalence of microsporidia infections in small ruminants seems necessary.

The present systematic review and meta-analysis showed that microsporidia infection, with particular emphasis on *E. bieneusi*, is more prevalent in sheep (17.4%) than in goats (16.3%). Most microsporidia species are able to infect the gastrointestinal tract, while some species occupy the urinary tract, hence being found in urine samples. In this meta-analysis, only one study examined the molecular prevalence of *Enc. cuniculi* in urine samples, which was negative for all samples [30].

Although most studies used the nested PCR technique, some studies used the PCR and real-time SYBR green techniques. The most important advantage of nested PCR compared to the other two methods is that it could detect low amounts of microsporidia due to its high specificity [50, 51]. Moreover, nested PCR with the ITS gene is able to identify different *E. bieneusi* genotypes [51], whereas PCR with SSU rRNA gene fails to identify genotypes [52]. Genotyping of *E. bieneusi* using ITS gene sequence has been the most preferred and the gold standard method in recent decades, offering adequate information on pathogenicity and source of the organism [53]. Reportedly, BEB6, COS-1, and CHG3 of *E. bieneusi* have been the most prevalent genotypes among ruminants, in particular sheep and goats [39, 42, 53]. Of note, other less common zoonotic genotypes (Peru 6 and I), were also found in the present review, mostly isolated from humans and small ruminants [53]. This indicates to the possible environmental transmission of infective spores between humans and small ruminants. However, many samples from these animals and humans should be genotyped to endorse the zoonotic transmission of the genotypes.

China possessed the largest dataset (24 datasets) with a pooled prevalence rate of 17.9%, while only 7 other countries had reported microsporidia infection in sheep and goats. Still little is known regarding microsporidian infections in small ruminants in many countries worldwide, particularly in those nations having traditional animal husbandry system. As shown in Table 2, some key countries have few studies which implicates the need for further studies and more attention to sheep and goats microsporidiosis in these countries. It is noteworthy that information derived from the Europe (three studies), Africa (two studies), and America (one study) must be interpreted cautiously, because of paucity of studies (Table 2). There are several risk factors involved in the distribution of the microsporidian agents, including climatic variation, type of animal husbandry, parasite control measures, Human Development Index (HDI), etc. [11, 54]. Traditional animal husbandry systems facilitate the access of small ruminants to other domestic, wild and stray animals or close contact with environmental sources (e.g., consumption of spores contaminated water and food) [1, 7, 20, 51]. As such, different animals, water resources, and vegetables play a crucial role in maintaining the microsporidia cycle. Therefore, sheep and goats may be considered as a major reservoir of microsporidia, which subsequently may be responsible for the outbreaks of human microsporidiosis.

In the present meta-analysis, we found a higher microsporidia prevalence in ≤ 3 months and male animals, being statistically significant. Younger animals have immature and/or deficient immune status, hence they may be more susceptible to the microsporidia infection [26, 27], as substantiated by the higher prevalence in this review.

This systematic review and meta-analysis has some limitations and the results presented here should be interpreted with respect to these limitations, comprising lack of prevalence information in many countries; low sample size in some studies; and lack of risk factor (i.e., age and gender) and clinical symptoms (i.e., gastrointestinal disorders) assessment in most studies. Moreover, although this is a global meta-analysis on the molecular prevalence of microsporidia in sheep and goats, only eligible published studies were included, and it is possible that useful data were missed from the ‘grey’ literature. Also, online registration in PROSPERO failed, because data were already extracted. Considering these limitations, it is noteworthy to say that our results may be not precisely reflect the true prevalence, and the presented numbers are apparent prevalence rates. Nevertheless, it is believed what we had reported here is close to true microsporidia prevalence in sheep and goats within a global context.

## Conclusion

This study showed a relatively high prevalence of microsporidia infection in sheep and goats worldwide, which could be directed towards better control and prevention of microsporidia infection in sheep and goats. Further, the findings of the present study should be taken into account by the health care authorities, physicians, veterinarians of the countries. The high-risk groups including immunocompromised patients must receive accurate and valid information about the risk of contact with the infected these ruminants. We suggest performing further studies to clarify the global prevalence of microsporidiosis based on molecular methods, which would be a guide to the establishment of appropriate public health interventions.

## Supplementary Information

**Additional file 1.Figure S1.** The pooled molecular prevalence of microsporidia infection in sheep. **Figure S2. **The pooled molecular prevalence of microsporidia infection in goats. **Figure S3.** Publication bias using funnel plot. Publication bias in sheep datasets. Funnel plot displaying prevalence data for all included publications (*n* = 20). Each circle represents reported prevalence from one individual study. Please note wide value distribution outside the funneled area indicating significant publication bias. **Figure S4.** Publication bias using funnel plot. Publication bias in goat’s datasets. Funnel plot displaying prevalence data for all included publications (*n* = 14). Each circle represents reported prevalence from one individual study. Please note wide value distribution outside the funneled area indicating significant publication bias.

## Data Availability

Not applicable.
