# Supplementary material for: The global molecular epidemiology of microsporidia infection in sheep and goats with focus on Enterocytozoon bieneusi: a systematic review and meta-analysis
Source: Trop Med Health. 2021 Aug 24;49:66. doi: 10.1186/s41182-021-00355-7 (PMC8385986; doi:10.1186/s41182-021-00355-7)
Supplement: Supplementary file 1 — Additional file 1.Figure S1. The pooled molecular prevalence of microsporidia infection in sheep. Figure S2. The pooled molecular prevalence of microsporidia infection in goats. Figure S3. Publication bias using funnel plot. Publication bias in sheep datasets. Funnel plot displaying prevalence data for all included publications (n = 20). Each circle represents reported prevalence from one individual study. Please note wide value distribution outside the funneled area indicating significant publication bias. Figure S4. Publication bias using funnel plot. Publication bias in goat’s datasets. Funnel plot displaying prevalence data for all included publications (n = 14). Each circle represents reported prevalence from one individual study. Please note wide value distribution outside the funneled area indicating significant publication bias. [file 41182_2021_355_MOESM1_ESM.docx]

**
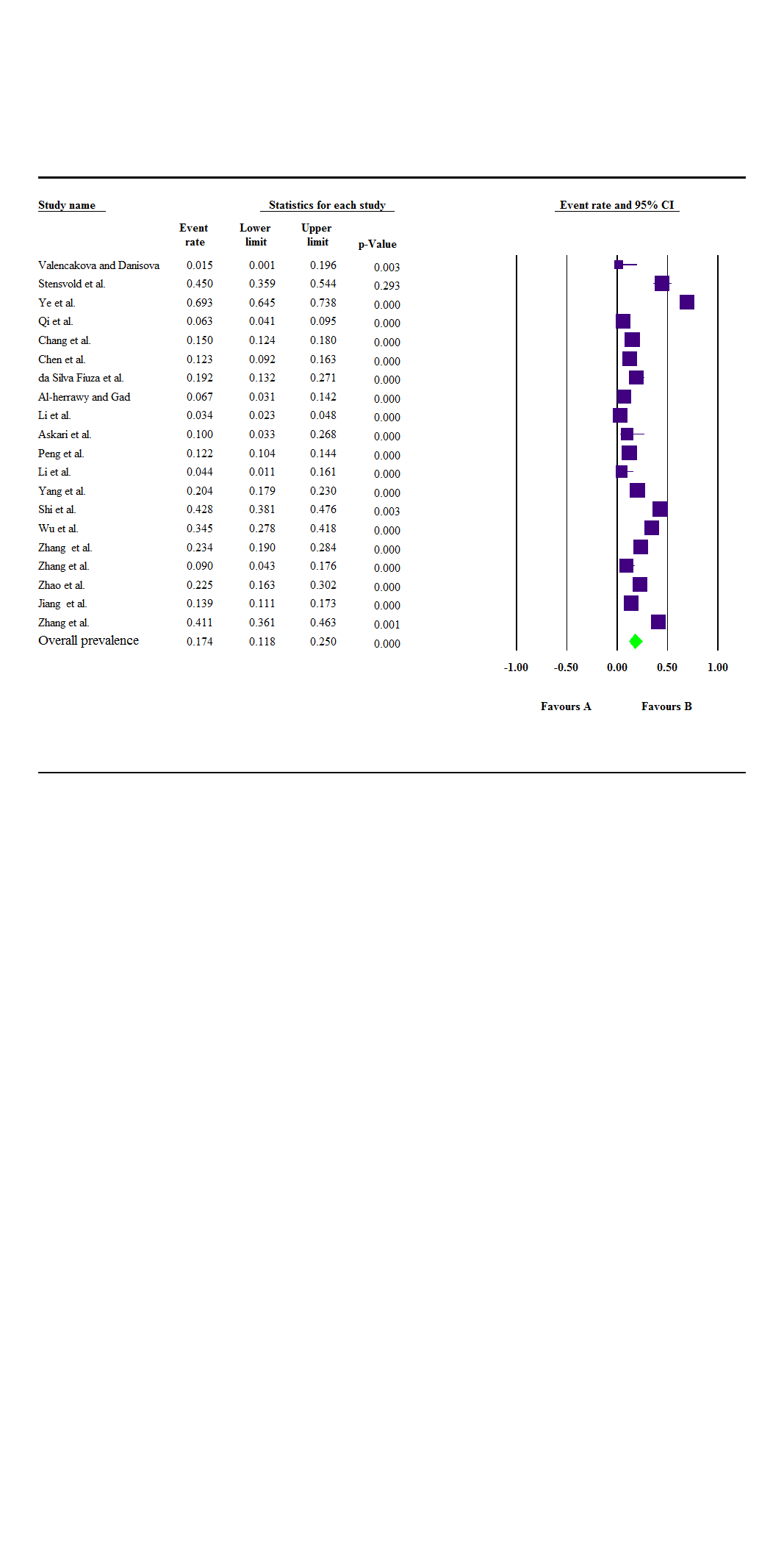
**

**Additional Figure S1.** The pooled molecular prevalence of microsporidia infection in sheep.


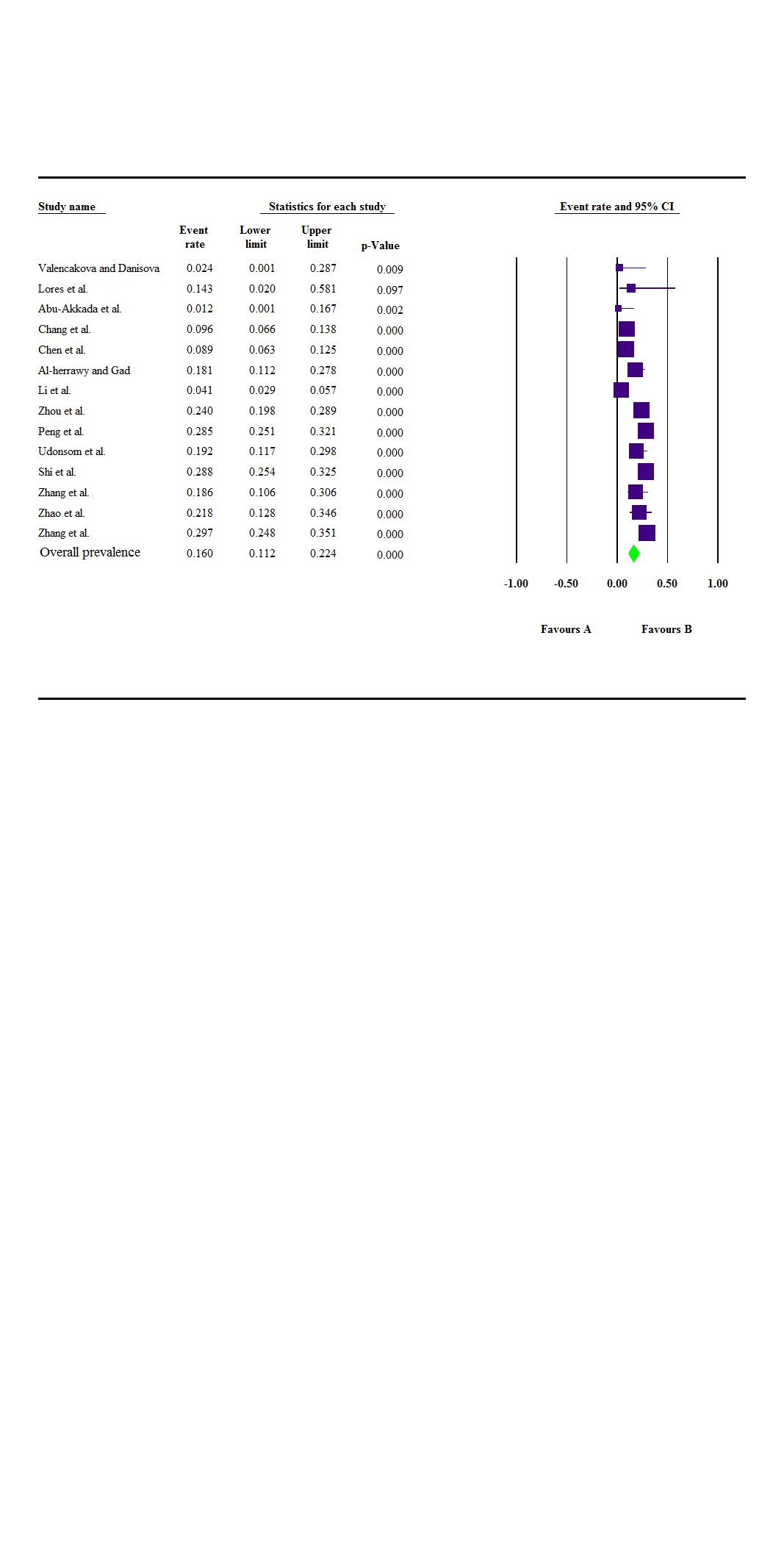


**Additional Figure S2.** The pooled molecular prevalence of microsporidia infection in goats.


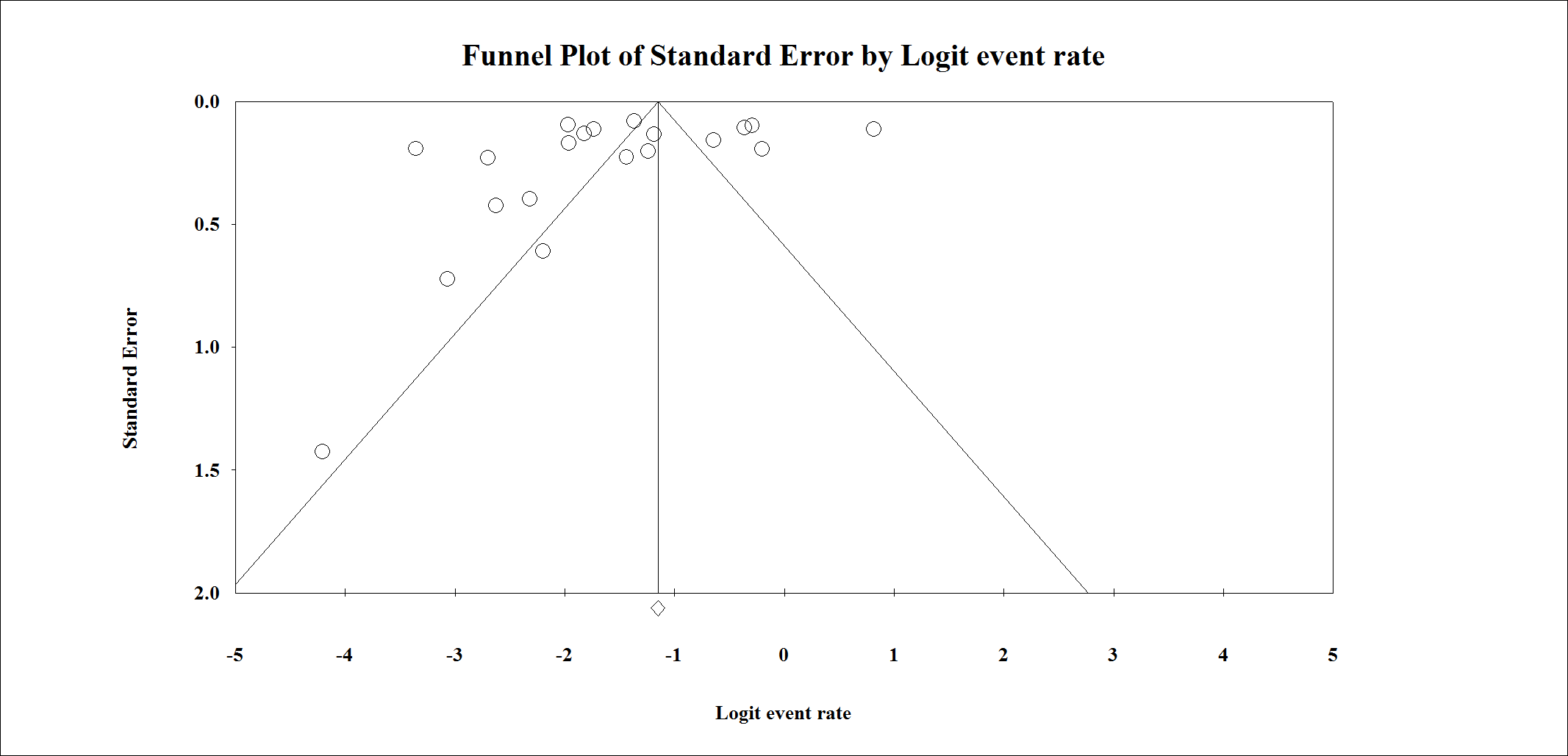


**Additional Figure S3. Publication bias using funnel plot.** Publication bias in sheep datasets. Funnel plot displaying prevalence data for all included publications (n = 20). Each circle represents reported prevalence from one individual study. Please note wide value distribution outside the funneled area indicating significant publication bias.


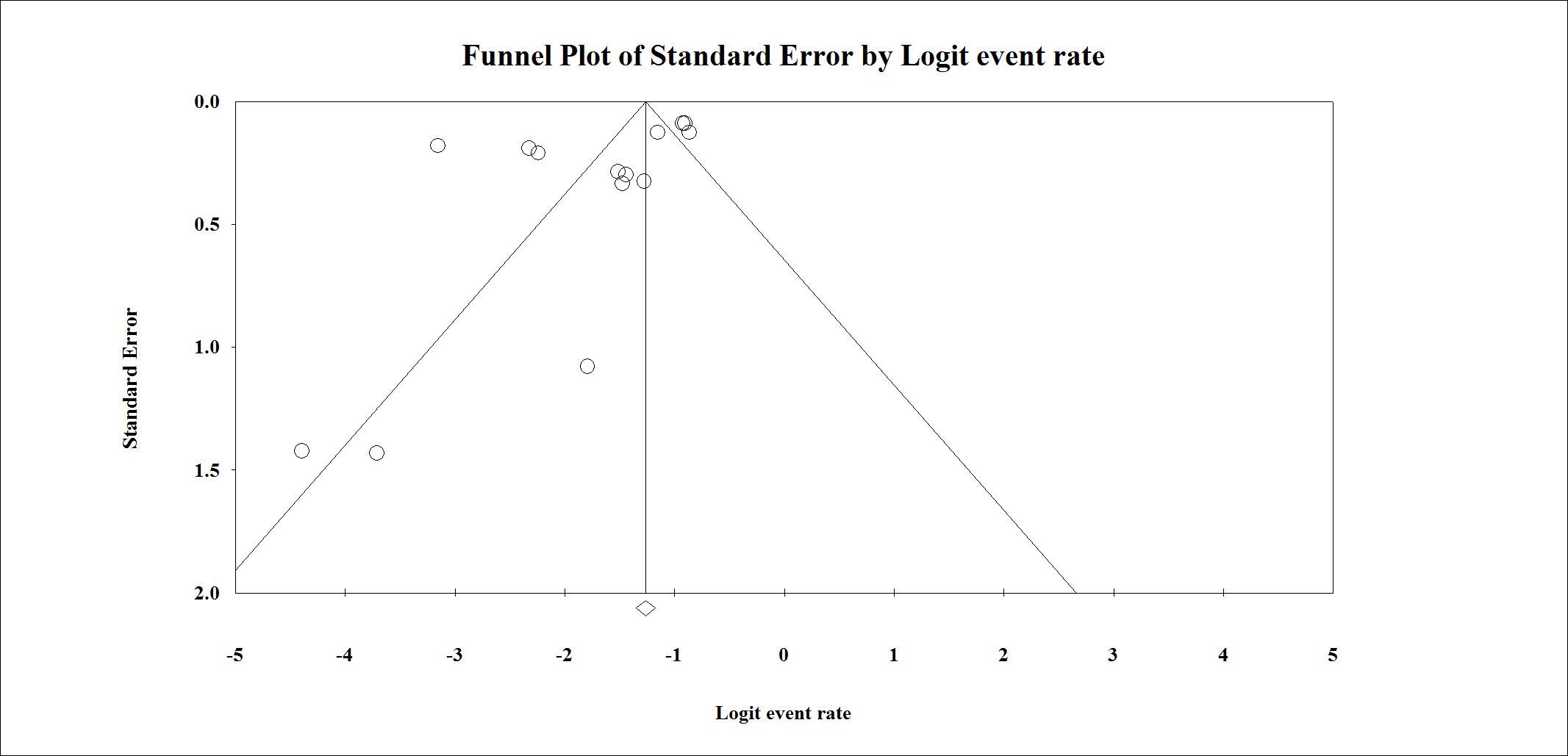


**Additional Figure S4. Publication bias using funnel plot.** Publication bias in goat’s datasets. Funnel plot displaying prevalence data for all included publications (n = 14). Each circle represents reported prevalence from one individual study. Please note wide value distribution outside the funneled area indicating significant publication bias.
